# Vertical transmission of Severe Acute Respiratory Syndrome Coronavirus 2: A scoping review

**DOI:** 10.1371/journal.pone.0250196

**Published:** 2021-04-22

**Authors:** Lemi Belay Tolu, Alex Ezeh, Garumma Tolu Feyissa

**Affiliations:** 1 Saint Paul’s Hospital Millennium Medical College, Department of Obstetrics and Gynaecology, Addis Ababa, Ethiopia; 2 Dornsife School of Public Health, Drexel University, Philadelphia, PA, United States of America; Mount Sinai Health System, University of Toronto, CANADA

## Abstract

**Introduction:**

The evidence for vertical transmission of Severe Acute Respiratory Syndrome Coronavirus 2 (SARS-CoV-2) is not well established. Therefore, the objective of this review is to summarize emerging evidence on the vertical transmission of Severe Acute Respiratory Syndrome Coronavirus 2.

**Methods:**

We conducted a systematic search in PubMed, CINAHL, Web of Science, SCOPUS, and CENTRAL. Likewise, a search for preprint publications was conducted using MedRxiv and Research Square. Studies that addressed vertical transmission of SARS-CoV-2 (concept) among pregnant women infected by Covid-19 (population) in any setting (community, hospital, or home) in any country or context were considered for inclusion. Any types of studies or reports published between December 2019 and September 2020 addressing the effects of SARS-CoV-2 on pregnant women and their newborn babies were included. Studies were screened for eligibility against the inclusion criteria for the review by two reviewers.

**Results:**

We identified 51 studies reporting 336 newborns screened for COVID-19. From the 336 newborns screened for COVID-19, only 15 (4.4%) were positive for throat swab RT-PCR. All neonates with positive throat swab RT-PCR were delivered by cesarean section. Among neonates with throat swab SARS-CoV-2 positive only five (33.3%) had concomitant placenta, amniotic fluid, and cord blood samples tested, of which only one amniotic fluid sample is positive for RT PCR. Five neonates had elevated IgG and IgM but without intrauterine tissue tested. Four neonates had chest imaging suggestive of COVID-19 pneumonia.

**Conclusion:**

Currently there is not enough evidence on vertical virologic transmission of COVID-19 infection during the third trimester of pregnancy. Additionally, there is no evidence to support cesarean delivery, abstaining from breast feeding nor mother and infant separation. Further research involving an adequate sample size of breast milk, placenta, amniotic fluid, and cord blood to ascertain the possibility of vertical transmission and breast milk transfer is needed.

## Introduction

Severe acute respiratory syndrome coronavirus 2 (SARS CoV-2), also known as COVID-19, is an ongoing pandemic worldwide [1]. The physical and physiological changes in pregnancy might increase the complications of some respiratory infections like SARS-CoV-2. However, limited data with a limited number of participants showed no difference between the clinical manifestations of COVID-19 pregnant and non-pregnant women or adults of reproductive age [2–4]. A major concern of SARS-CoV-2 infection is vertical maternal-fetal transmission. Limited data from case reports and case series suggest no evidence of mother-to-child transmission when infection manifests in the third trimester of pregnancy [5]. A systematic review of 22 studies (case reports and case series) did not support intrauterine vertical transmission of SARS-CoV-2 [6].

As the pandemic is new and evolving, the evidence available to date remains limited. Available studies and reports are mostly small in sample size. Pooling these studies and reports together might increase the power in identifying important patterns. Therefore, this scoping review was aimed to identify, map, and describe reports and studies that addressed vertical transmissions in pregnant women infected by COVID-19. The findings may provide valuable insight into the vertical transmission of SARS-CoV-2. Hence, it may lay the ground for future more rigorous research.

## Review question(s)

What evidences are available regarding the vertical transmission of SARS-CoV-2? Are there possibilities for vertical transmission of SARS-CoV-2?

## Methods

We reported the review following Preferred Reporting Items for Systematic Review and Meta-Analysis (PRISMA-Sc) guidelines–extension for scoping review (S1 Table) [7]. The review was conducted per the Cochrane handbook for a systematic review [8]. During the conduct of the review, we considered the following inclusion criteria:

### Participants

We considered pregnant women infected by SARS-CoV-2 confirmed by SARS-CoV-2 by RT-PC laboratory and newborns delivered by SARS-CoV-2-infected mothers.

### Concept

We considered studies addressing the vertical transmission among pregnant women diagnosed with SARS-CoV-2 by RT-PCR (laboratory-confirmed diagnosis). The potential impact may include but not be limited to vertical transmission. The study bio-specimens included cord blood, amniotic fluid, placenta, vaginal secretion fluid and breast milk.

### Context

The review considered any report in any community or healthcare setting in any country.

### Study type

In this review, we did not restrict the reports based on study design. Rather, we considered all studies including, but not limited to randomized trials, before and after studies, case reports, case-control, cross-sectional studies, cohort studies, case series, letters to the editor, and short communications published in preprints and peer-reviewed journals. We included studies published or reported between December 2019 and September 2020.

### Search strategy

The search strategy aimed to locate both published and unpublished studies. We initially conducted a limited search in MEDLINE to identify articles on the topic. The text words contained in the titles and abstracts of relevant articles, and the index terms used to describe the articles were used to develop a full search strategy (see S1 Appendix search strategy). We conducted a systematic search in PubMed, CINAHL, Web of Science, SCOPUS, and CENTRAL. We conducted a further search for preprint publications in MedRxiv and Research Square. Besides, we screened the reference list of all selected studies for additional studies.

### Study selection

Following the search, we collated and uploaded all identified citations into EndNote X8 (Thomson Reuters, USA) and removed duplicates. Two independent reviewers screened the records against the inclusion criteria for the review. We recorded the reasons for the exclusion of full-text studies that did not meet the inclusion criteria. Any disagreements between the reviewers at each stage of the study selection process were resolved through discussion. We reported the results of the full systematic search and presented in a Preferred Reporting Items for Systematic Reviews and Meta-analyses (PRISMA) flow diagram [9].

### Data extraction and synthesis

Two reviewers recorded the data using a formatted electronic data abstraction form. We extracted the data on the study author, country, study populations, study design, gestational age, mode of delivery, newborn feeding practice, laboratory samples taken and their result, maternal disease severity and maternal and foetal outcome. Any disagreements between the reviewers were resolved through discussion, or with a third reviewer. Authors of papers were contacted to request missing or additional data, where required. We organized and summarized results as clinical presentation, pregnancy status, maternal and perinatal outcomes using frequency and percentages. We did not perform a formal critical appraisal of individual studies for this systematic scoping review.

## Results

**Fig 1:**
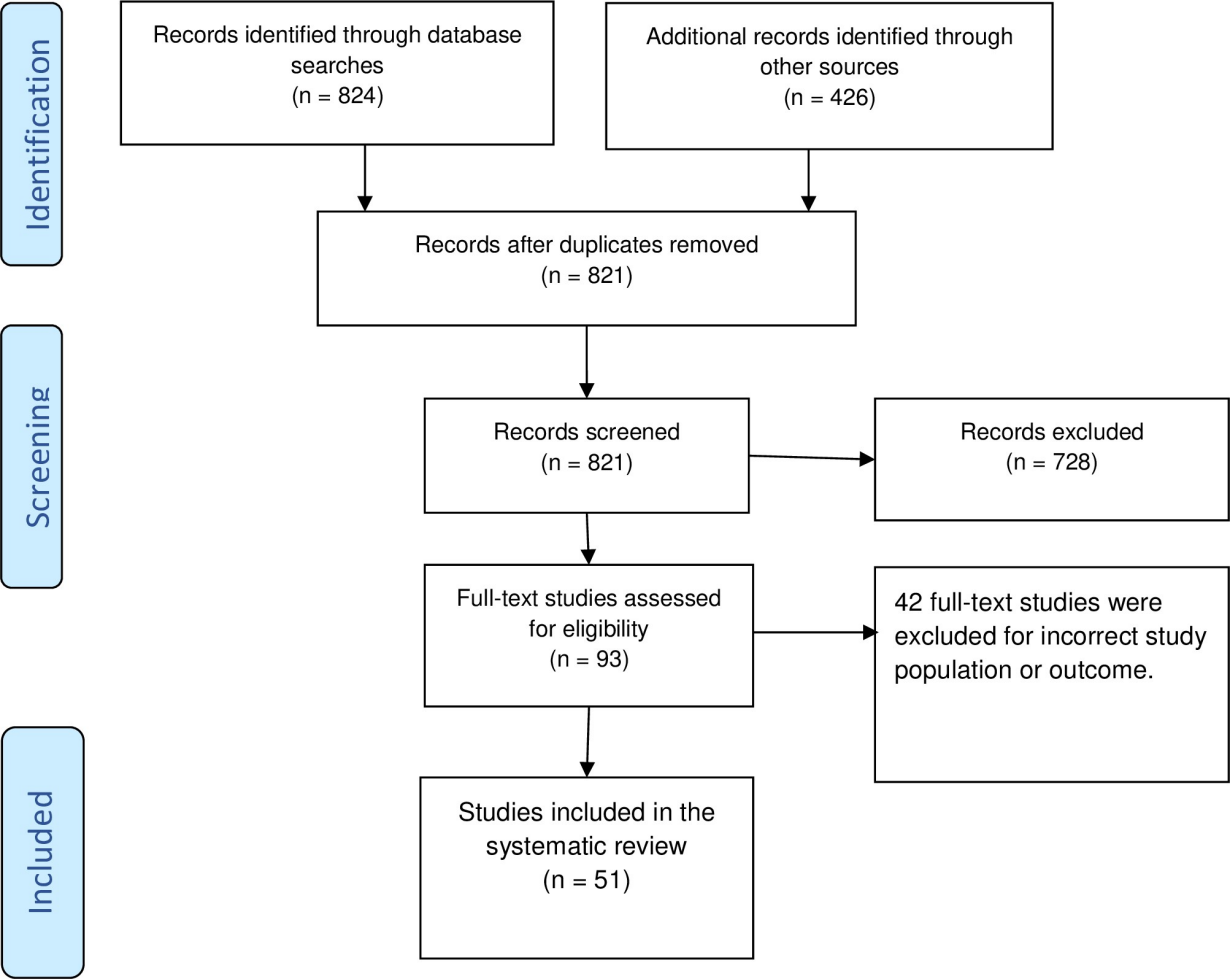
The Prisma flow diagram illustrating the study selection process.

The initial search yielded 1250 records. After removing duplicates, we screened the title and abstract of 821 records against inclusion criteria. We did a full-text review of 93 studies, and excluded 42 studies, and retained 51 studies reporting on 336 newborns for scoping review (see Fig 1). Of the 51 studies, 30 were case series, 20 case reports, and one was a case-control study. Among these studies, 37 were from China [10–44], 5 were from the USA [45–49], 3 were from Italy [50–52], and one study was reported from each of the following countries: Iran [53], Australia [54], Belgium [55], Korea [56], Sweden [57] and Turkey [21] (see Table 1).

**Table 1:** Types of studies included in the review.

Study design	Country	Count of studies	COVID-19 cases
Case-control	China	1	16
Case reports	China	9	9
	USA	4	4
	Italy	1	1
	Sweden	1	1
	Belgium	1	1
	Korea	1	1
	Australia	1	1
	Iran	1	1
	Turkey	1	1
Case series	China	27	287
	USA	1	2
	Italy	2	11
Total		51	336

### Disease severity and delivery status

Among the 336 newborns screened for COVID-19, the maternal disease was reported as mild in 301 (89.5%), severe in 26 (7.7%), and critical in 9 (2.6%) of the cases. All maternal COVID-19 was confirmed by SARS-CoV-2 RT-PCR. All 336 screened newborns were singletons, 40 (11.9%) births were preterm (<37 weeks) and all were live births except for one stillbirth at 20 weeks. Among the preterm births, 18 (45%) of them were spontaneous preterm labour, while 17 (42.5%) were indicated preterm birth for COVID-19, and 5 (12.5%) were indicated preterm birth for obstetric indications. Among the 336 births, 265 (78.8%) were delivered by cesarean section, 55 (16.3%) vaginally, and 16 (4.7%) with the mode of delivery unknown.

Birthweight varied from 1600 g to 4100 g, and 21 (6.25%) of them were low birth weight (<2500g). Twenty-eight (8.3%) required admission to the neonatal intensive care unit (NICU). Among the 28 NICU admissions, 12 (42.8%) were preterm and 16 (57.1%) were term births. Admission diagnosis to NICU was respiratory distress syndrome in 7 (25%), neonatal pneumonia in 9 (32.1%), perinatal asphyxia in 2 (7.1%), and unknown in 10 (3.6%).

### Vertical transmission

In the current review, we identified 51 studies reporting on 336 newborns screened for COVID-19 from which 15 (4.4%) were positive (Table 2), and 321 (95.6%) were negative for throat swab SARS-CoV-2 RT-PCR. RT-PCR SARS-CoV-2 tests were also done on 108 cord blood, 111 amniotic fluid, 53 breast milk, 20 vaginal secretions, and 15 placenta samples. All those samples were negative except for one placenta sample and one amniotic fluid sample which were positive. In 15 antibody tests, five neonates had both IgG and IgM elevated, six neonates had elevated IgG and normal IgM, and 4 had both IgG and IgM normal (Table 2). All 15 neonates with positive throat swab RT-PCR were delivered by cesarean section. Among the throat swab RT-PCR positive neonates, the test sample was taken immediately after delivery for 2 (13.3%), at 24 hrs for 5 (33.3%), at 16 hours for 2 (13.3%), at 36 hours for 3 (20%), at 6^th^ day for 2 (13.3%) and one week for 1 (6.6%). Among the neonates with a positive result for SARS-CoV-2, only one (6.6%) had contact with the mother with expressed breast milk (Table 2). Among the neonates with throat swab SARS-CoV-2 positive, only five (33.3%) had concomitant intrauterine tissue tested: two had amniotic fluid and cord blood tested, two had placenta and cord blood and one had amniotic fluid tested. All concomitant intrauterine tissues tested were negative except for the study of Zamaniyan et al. [53] in which amniotic fluid test was positive. Four of the neonates had chest imaging suggestive of COVID-19 pneumonia (Table 2). David Baud et al. reported on a 19 weeks stillbirth with placenta membranes and cotyledon positive for SARS-CoV-2 but amniotic fluid, cord blood, liver, fetal mouth, and thymus tissue all negative [58].

**Table 2:** Characteristics of studies reporting on the possibility of vertical transmission of SARS-CoV-2.

Author and country	Gestational age	Mode of delivery	Type of sample and test result	Sampling time after birth	Feeding	Mother-infant isolation?
Alzamora et al. [45] USA	33 weeks	Cesarean section	SARS-CoV-2 RT-PCR positive IgG and IgM negative CxR normal Amniotic fluid, placenta cord blood not tested	16 hrs	Formula feeding	Yes for 14 days
Xiaolin Hu [20] China	40 weeks	Cesarean section	SARS-CoV-2 RT-PCR positive CxR normal Amniotic fluid test negative, no other test.	16 hrs	Formula-feeding	Yes for 14 days
Liu W et al. [26] China	38 weeks	Cesarean section	SARS-CoV-2 RT-PCR positive Amniotic fluid and cord blood negative, others not done		Formula feeding	Yes for 14 days
Khan S et al. [23] China	37 and 38 weeks	Cesarean section	2 neonates SARS-CoV-2 RT-PCR positive, intrauterine tissues not tested.	24 hrs	Formula feeding	Yes for 14 days
Nie Rui et al. [29] China	Not specified	Cesarean section	SARS-CoV-2 RT-PCR positive CXR-pulmonary infection Placenta and cord blood negative, others not done	36 hrs	Formula feeding	Yes for 14 days
Piersigilli et al. [55] Belgium	27 weeks	Cesarean section	SARS-CoV-2 RT-PCR positive CXR-pulmonary normal Intrauterine tissue tests not done, breast milk negative	7^TH^ DAY	Expressed breast milk	Together for the first 24 hrs
Sun Mingyang et al. [32] China	36 and 37 weeks	Cesarean section	1 neonate SARS-CoV-2 RT-PCR positive, 1 neonate chest CT GGO (clinical but PCR negative), Intrauterine tissue test not done for both	6^th^ day	Formula feeding	Yes for 14 days
Wang S et al. [33] China	40 weeks	Cesarean section	SARS-CoV-2 RT-PCR positive Placenta and cord blood negative, others not done	36 hrs	Formula feeding	Yes for 14 days
Yu Nan et al. [39] China	37 weeks	Cesarean section	SARS-CoV-2 RT-PCR positive Chest x-ray-mild pulmonary infection, Intrauterine tissue tests not done	36 hrs	Formula feeding	Yes for 14 days
Zamaniyan Marzieh et al. [53] Iran	32-weeks	Cesarean section	Immediate first SARS-CoV-2 PCR negative, amniotic fluid positive, cord blood negative, 2^nd^,3^rd^, and 4^th^ throat swab positive	Immediate, 24 hrs and one week	Formula feeding	Yes for 14 days
Zeng Lingkong et al. [41] China	2 at 40 weeks 1 at 31 weeks	All Caesarean section	3 neonates SARS-CoV-2 PCR positive and CXR showed pneumonia 2 neonates had increased IgG and IgM, 3 had increased IgG and normal IgM, intrauterine tissue test not done	24 hrs	Formula feeding	Yes for 14 days
Dong Lan et al. [17] China	34 weeks	Cesarean section	IgG and IgM elevated Chest CT immediate normal and 7^th^-day GGO, throat swab not done, intrauterine tissue tests not done Breast milk PCR negative	Immediate and 7^th^ day	Formula feeding	Yes for 14 days
Zeng Hui et al. [40] China	Not specified	Cesarean section	2 neonates IgG and IgM elevated, 3 neonates had elevated IgG and normal IgM, all blood and throat swab PCR negative, intrauterine tissue not tested.	Immediate	Formula feeding	Yes for 14 days
David Baud et al. [58] Switzerland	19 weeks	Vaginally	Amniotic fluid, cord blood, fetal mouth, liver, thymus, and lung are negative. Placenta membranes and cotyledon positive for SARS-CoV-2	Immediate	Stillbirth	Stillbirth

CXR-Chest X-ray, GGO- ground-glass opacity.

## Discussion

This scoping review identified 51 studies reporting 336 newborns screened for SARS-CoV-2. From the 336 newborns screened for COVID-19, only 15 (4.4%) were positive for throat swab RT-PCR. All neonates with positive throat swab RT-PCR were delivered by cesarean section. Among the neonates with throat swab SARS-CoV-2 positive, only five (33.3%) had concomitant intrauterine tissue tested. All concomitant intrauterine tissues tested were negative except for the report of Zamaniyan et al. [53] with amniotic fluid testing positive. Five neonates had elevated IgG and IgM but without intrauterine tissue teste. Therefore, there is no adequate evidence suggesting the presence of vertical transmission of SARS-CoV-2. The current review finding is similar to other systematic reviews on vertical transmission of COVID-19 [59–64]. However, the current review is specific to the vertical transmission and included more studies. Besides, the review included only newborns born to mothers with laboratory-confirmed COVID-19. Follow up of laboratory-confirmed maternal infection with subsequent intrauterine tissue and newborn screening is important to reduce confounding due to misdiagnosis of infection and determine exactly when the transmission might have occurred. The positive throat swab RT-PCR in the newborns might be a horizontal transmission with postpartum infection from health care workers or their mothers unless confirmed by intrauterine tissue sample tests. Consequently, there is a need for caution in the interpretation of newborn data concerning when the viral transmission occurred.

Furthermore, the current review indicates that in all neonates with positive RT PCR, 54% were separated from their mother and did not breastfeed. This may be because of the fact that most of the studies were from China; the Chinese Expert Consensus on the Perinatal and Neonatal Management for the Prevention and Control of the 2019 Novel Coronavirus Infection [65] recommends that symptomatic pregnant women should be isolated. However, the breastmilk of 53 mothers tested negative for SARS-CoV-2 which is reassuring for breastfeeding mothers, and health care professionals to implementing the WHO recommendations of breastfeeding, especially in resource limited settings [66].

In this scoping review, we tried to capture studies published in preprints and peer-reviewed journals to have as many studies as possible for the evidence synthesis. However, the current review has some limitations. The pandemic is an evolving issue and our search addressed articles published by September 8, 2020. Besides, most of the studies included are case series and case reports, making it difficult in drawing firm conclusions. Nevertheless, with the above limitations in mind, the scoping review provides important insights into the potential vertical transmission of SARS-CoV-2. Such preliminary evidence might encourage more rigorous research for more solid evidence to inform clinical decisions.

## Conclusions

### Implications for practice

Currently, there is not enough evidence on vertical virologic transmission of COVID-19 infection in the third trimester of pregnancy. All neonates with positive RT PCR were born by cesarean section indicating that the cesarean section may be not necessarily safer than vaginal delivery in preventing mother-to-child transmission of COVID-19. This is in line with the WHO recommendation on the mode of delivery which should be based on obstetric indications. Given the lack of a virus in the breast milk samples, the current evidence does not support the abstaining of breastfeeding, nor mother and newborn separation. However, the implementation of strict infection prevention and control measures and monitoring breast feeding infants is essential.

### Implications for research

Evidence on vertical transmission of SARS-CoV-2 is limited to case reports and case series with no adequate intrauterine tissue samples tested. Therefore, we recommend further research test adequate breast milk, placenta, amniotic fluid, and cord blood samples to ascertain or disapprove the possibility of vertical transmission of COVID-19 and transmission through breast milk. In addition, there is a need for further research to determine long term outcomes of SARS-CoV-2 infection during pregnancy. The long-term consequences of COVID-19 infection among newborns who acquired the infection during or after delivery should be further investigated.

## Supporting information

S1 Table The PRISMA checklist. (DOCX)

S1 Appendix Search strategy. (DOCX)
