# Supplementary material for: Vertical transmission of Severe Acute Respiratory Syndrome Coronavirus 2: A scoping review
Source: PLoS One. 2021 Apr 22;16(4):e0250196. doi: 10.1371/journal.pone.0250196 (PMC8062014; doi:10.1371/journal.pone.0250196)
Supplement: S1 Appendix — (DOCX) [file pone.0250196.s002.docx]

**S1 Appendix. Search strategy.**

| Number | Search Query | Result. |
| --- | --- | --- |
| 1 | coronavirus[tw] OR COVID-19[tw] OR coronavirus [Mesh] AND gestation[mesh] OR pregnan*[tw]AND Neonate [Mesh] OR Neonat*[tw] | 415182 |
| 2 | Limit 1 to English AND Human AND 1 year. | 727 |

**CENTRAL**

([mh gestation] OR [mh Neonate] OR Neonat*:ti,ab,kw OR pregnan*:ti,ab,kw) AND ([mh coronavirus] OR "covid 19":kw OR COVID-19: ti,ab,kw OR COVID19:ti,ab,kw OR coronavirus:ti,ab,kw)

**Web of Science**

(gestation OR Neonate OR Neonat* OR pregnan*) AND (coronavirus OR "covid 19" OR COVID-19 OR COVID19 OR coronavirus) NOT (animals NOT humans)

**CINAHL**

((MH "gestation+") OR (MH "Neonate+") OR Neonat* OR pregnan*) AND ((MH "coronavirus+") OR MW "covid 19" OR COVID-19 OR COVID19 OR coronavirus) NOT ((MH "animals+") NOT (MH "humans+"))

**SCOPUS**

(INDEXTERMS("gestation") OR INDEXTERMS("Neonate") OR TITLE-ABS-KEY("Neonat*") OR TITLE-ABS-KEY("pregnan*")) AND (INDEXTERMS("coronavirus") OR CHEM ("covid 19") OR TITLE-ABS-KEY("COVID-19") OR TITLE-ABS-KEY("COVID19") OR TITLE-ABS-KEY("coronavirus")) NOT (INDEXTERMS("animals") NOT INDEXTERMS("humans"))

**MedRxiv and Research Square**

Search terms; Pregnancy, Pregnant, Coronavirus, SARS, Severe acute respiratory syndrome, MERS, Middle East Respiratory Syndrome, HCov-229E, HCov-NL63, HCov-OC43, HCov-HKU1
